# Chemical Composition and Enzymatic Screening of* Micromeria fruticosa serpyllifolia* Volatile Oils Collected from Three Different Regions of West Bank, Palestine

**DOI:** 10.1155/2018/6536919

**Published:** 2018-10-16

**Authors:** Nihaya Salameh, Naser Shraim, Nidal Jaradat

**Affiliations:** Department of Pharmacy, Faculty of Medicine and Health Sciences, An-Najah National University, Nablus, P.O. Box 7, Nablus, State of Palestine

## Abstract

**Introduction:**

Volatile oils (VOs) have been commonly used in cosmetics and food as fragrances, flavoring, and preservative agents or in alternative medicine for their therapeutic effects. This necessitates investigating those plants and their VOs. This study was conducted to investigate the chemical compositions of the VOs of* Micromeria fruticosa serpyllifolia* growing widely in three regions in Palestine (i.e., Hebron, Ramallah, and Nablus districts representing south, middle, and north of West Bank). Afterwards, VOs were subjected to* in vitro* screening and their enzymatic properties were compared.

**Methods:**

The analysis of chemical components of VOs was performed by gas chromatography coupled with mass spectrometry (GC-MS). The antilipase activity was evaluated using porcine pancreatic lipase and* p*-nitrophenyl butyrate. The antiamylase activity was assessed using porcine pancreatic *α*-amylase, starch, and 3,5-dinitrosalicylic.

**Results:**

Plant extracts yield range was 0.67 to 0.99 w/w%. GC-MS analysis showed the high percentages of oxygenated components in the range of 86.1-89.88% and nonoxygenated components in the range of 4.38-4.71%. Seven components were observed, pulegone was the most abundant component in the three samples in the range of 74.43-86.04%, and isomenthone was the second most abundant component with the range of 3.16-14.41%. The sample collected from Nablus showed the most potent antilipase and antiamylase activity with IC50 values of 39.81 *μ*g/mL and 3.31 *μ*g/mL, respectively.

**Conclusions:**

The study showed that* Micromeria fruticosa serpyllifolia* volatile oils samples from different regions in Palestine contained different proportions of phytochemicals which provided different potential biological activities such as antiobesity and antidiabetes activities that were in line with traditional uses of the plant extracts. The plant extracts showed higher antilipase and antiamylase potency than that of the relative references and there were significant differences in these activities compared to each other.

## 1. Introduction

Dissimilar to conventional single drug, plant extracts or raw plants have a range of phytochemicals and bioactive constituents that provide synergistic effects which allow for multitarget effect in curing of diseases [1]. The medicinal plants and their claimed traditional use are considered one of the major approaches in developing new drug from natural products [2]. Secondary metabolites, including alkaloids, glycosides, flavonoids, phenols, steroids, saponins, tannins, terpenoids, and volatile oils (VOs), are important for healing diseases and are responsible for the therapeutic effect of plants; for example, VOs have anti-inflammatory, anticancer, anthelmintic, antimalarial, antiviral, antibacterial, cholesterol inhibition, and insecticide effects [3, 4]. Volatile oil also called “ethereal oil” or “essential oil” is extracted from different parts of plant (roots, bark, leaves, flowers, fruits, etc.) [5]. The chemical compounds of VOs can be classified into oxygenated (ketones, alcohols, phenols, etc.) and hydrocarbons (limonene, pinene, etc.) [6, 7]. The chemical structures of VOs determine their therapeutic activities [6]. The chemical composition and the aroma of VOs may be different due to growing condition (climate, type of soil and composition, altitude), plant age, geo-climatic location, and environmental conditions of collection time and site [8].

Dangerous health problems causing big load to global health sector such as obesity are a global health danger, which negatively affect personal professional quality of life, morbidity, and mortality [9]. The greatest cost expended for obesity is due to coronary heart disease, diabetes type 2, and hypertension [10]. Orlistat is among the most common drugs used for obesity treatment, but there is still deficiency of safe medicine for treating obesity [9]. Diabetes has caused a major load to the global health sector [11]. Diabetes is a dangerous disease and has serious complications; WHO reported that 8.5% of adults around the world have diabetes, and 1.6 million deaths occurred in 2015 [12]. Therefore, the investigation for antidiabetic agents from plant extract has increased, as discovering new effective drugs is important for controlling the disease [11].

*Micromeria fruticosa* subspecies* serpyllifolia* (M. Bieb.) (Lamiaceae), known as White* Micromeria*, is an aromatic herb [13], dominated in the eastern Mediterranean regions including Palestine, has pleasant minty fragrance, and in hot summer provides sensation of coolness [14, 15]. In Palestinian society known as Duqat ‘Adas, ‘Ishbit esh-shai, Qurnya [16, 17], and as Thyme-leave savory in English, the aerial parts of plant (flower, leaves, and stalk) are used in folk medicine [16].* M fruticosa serpyllifolia* is a perennial Mediterranean plant habitant in rocky areas that has a height of 20-80 cm. It is short-shrub plant grown in the period of end winter and spring and starts flowering in summer until autumn with white color [15, 18–20]. Stems are straight, whitish, covered with short, dense, and soft hair, thick, and solid. Leaves are greyish-white, thyme-leaved, and covered with very finely hair. Inflorescence is a cluster of cymes with many branching flowers. Corolla is yellow or white, scarcely female, and self-pollinated in unopened flowers [21–23].* M fruticosa serpyllifolia* has different uses in traditional medicine such as treatment of hypertension, heart disorders, diarrhea, abdominal pains, colds, headache, wounds, infections such as skin and eye infections and has anti-inflammatory effects [15, 24–28]. In Palestinian society* M fruticosa serpyllifolia* is considered one of the most wild edible plants in Palestine [14]. The leaves are prepared as tea for colds and relieve intestine and stomach pain in addition to exhaustion and weariness [15]. The extracts of leaves have been used for relief chest, respiratory system, asthma, fever, skin infections, wounds, and eye inflammation [18, 20]; in addition to that, the infusion of* M fruticosa serpyllifolia* stalks and leaves in Palestinian society is used in treatment of diabetes, cough, headaches, and urinary diseases [16, 20]. The major constituents of* M fruticosa serpyllifolia* VOs were monoterpenes (pulegone, menthol, isomenthol, isomenthone, limonene, *α*-pinene, *β*-pinene, piperitone, and piperitenone oxide), and sesquiterpenes (b-caryophyllene and germacrene) [15, 19, 24]. Studies conducted on the aqueous extract of* M fruticosa serpyllifolia* showed anti-inflammatory effect and protection against gastric ulcer activities so it can be used as supplement or alternative herbal therapy for NSAIDs which can cause gastric ulcers [18, 25].* M fruticosa serpyllifolia* VOs also exhibit antibacterial, antifungal, antioxidants, insecticide, analgesic, anticonvulsants, hepatoprotective, and Central Nervous System (CNS) depressant effects [13, 15, 26, 29].

They can also be used as a natural substance for replacement of synthetic herbicides due to the presence of pulegone which consisted of 70% of oils chemicals [30]. Therefore, the aim of the current study was to identify the chemical composition and the potential enzymatic activities of* M fruticosa serpyllifolia* growing wildly in three regions in West Bank area in Palestine and to perform a comparative study of the results among three regions in Palestine.

## 2. Materials and Methods

### 2.1. Chemical Reagents

Porcine pancreatic lipase, Tris-HCl, PNPB (*p*-nitrophenyl butyrate), Amylase type VI -B, ≥ 10 unit/mg, Acarbose, and Calcium Chloride were purchased from Sigma-Aldrich, USA. Orlistat was purchased from Sigma-Aldrich, China, Acetonitrile and dimethyl sulfoxide (DMSO) were purchased from CARLO ERBA, France. 3,5-Dinitrosalysylic acid (DNSA) was purchased from Sigma-Aldrich, India, sodium potassium tartrate tetrahydrate was purchased from Merck, Germany, sodium hydroxide (NaOH) was purchased from Sun Pharm.drug stars, Nablus, Palestine, Disodium hydrophosphate/dihydrosodium phosphate (Na_2_HPO_4_/NaH_2_PO_4_) was purchased from Alfa Aesar, USA, Sodium chloride (NaCl) was self-backing (Alshela company, Palestine) as well as Starch (Alzahra company, Nablus, Palestine).

### 2.2. Instrumentation

Grinder (Moulinex model, Uno. China) was used to fracture the dried herbs. Balance max 220 g (Radway, Poland) was used to weigh the plant material, microwave-ultrasonic cooperative extractor/reactor (CW-2000, China) was utilized for extraction volatile oil, and Gas Chromatography Mass Spectrometry (QP-5000 Shimadzu GC-MS, Japan) was utilized for chemical screening of VO. Balance was 4500 g (BOECO, Germany), Ultraviolet-Visible (UV-Vis) Spectrophotometer (Jen WAY 7315, UK) was utilized for assessment the enzymatic activities of VOs, water bath was from Memert, Germany, water bath sonicator was from MRC, Haifa, and heater was from Lab-Tech, Korea. pH meter was used to adjust pH of disodium hydrophosphate/dihydrosodium phosphate (Na_2_HPO_4_/NaH_2_PO_4_). Micropipettes were from Microliter, BRAND, Germany.

### 2.3. Plant Material Collection and Treatment

The aerial parts of* M fruticosa serpyllifolia *were collected in April of 2017 from three cities in the West Bank (WB) in Palestine: Nablus, Ramallah, and Hebron which represented north, middle, and south regions of the WB in Palestine, respectively (three samples were collected from each city). The samples were botanically identified and coded by Dr. Nidal Jaradat the Pharmacognosist at An-Najah National University (ANNU) under the voucher specimen code: Pharm-PCT-1575. The extraction of VOs followed the procedure in [31]. The fresh aerial parts of* M fruticosa serpyllifolia* were separated carefully, washed two times with distilled water, and dried for two weeks in the shade at room temperature. The dried specimens were fractured and stored in well closed plastic bags for future use in the Laboratory of Pharmacognosy at An-Najah National University Faculty of Medicine and Health Sciences.

### 2.4. Extraction of Volatile Oils

The VOs of the three samples of* M fruticosa serpyllifolia* plant were extracted by microwave-ultrasonic method which was examined by Jaradat* et al*, 2016 by which the suspension of plant fractures was exposed to ultrasonic waves to improve the extraction process [31]. The apparatus consisted of a microwave oven combined with an ultrasonic extractor. Approximately 100 g of the fractioned dried aerial parts of each plant sample was placed in a one litter round-bottom flask, about 300 mL deionized water was added, and the flask was placed in the apparatus connected with Clevenger apparatus placed in the same apparatus. The power of the microwave-ultrasonic extractor apparatus was fixed at 1000 W. The ultrasonic power of the apparatus was fixed at 50 W and the frequency of 40 kHz at its maximum power. The extraction process was prolonged for 10 min at 100°C. This was repeated three times for each plant sample. The resulting VOs were collected into a separate clean, well closed small glass bottle, chemically dried over calcium chloride, and stored in the refrigerator at 2-8°C until use [31, 32].

### 2.5. GC-MS Analysis

The chemical composition of the three samples of* M fruticosa serpyllifolia* VOs was separated and quantified using Shimadzu QP-5000 GC-MS. The method used was described by Mohammad Al-Hamwi* et al*. [13] and Jaradat* et al* [32] with some modifications. GC was equipped with column Rtx-5ms (0.25 mm inner diameter, 0.25*μ*m thickness, and 30 m length). A carrier gas was helium at a flow rate of 1 mL/min. The temperature of the injector was 220°C. The temperature of the oven was programmed from 50°C (1min hold) at 5°C/min to 130°C and then at 10°C/min to 250°C and kept constant for 15 min. The temperature transfer line was 290°C. For GC-MS detection, an electron ionization method, with detector volts of 1.7 KV, was utilized. A scan speed 1000 amu/sec and scan rate of 0.5 s were used, covering a mass range from 38 to 450 M/Z [13, 32].

### 2.6. Components Identification

The mass spectrometry data center of the national institute of standards and technology (NIST) was used as a reference to identify the chemical components of the VOs by comparing their MS spectra with data of NIST in addition to using Kovats index in the literature to compare their retention times. The quantitative data were obtained electronically from integrated peaks, area percentages without the use of correction factor [32, 33].

### 2.7. Pancreatic Lipase (PL) Inhibition

The porcine pancreatic lipase (PPL) inhibitory assay was conducted using the methods from Jaradat* et al*. [34], Bustanji* et al*. [35], and Siew-Ling* et al*. [36] with some modifications. VOs stock solution of 1mg/mL was prepared in 10% Dimethyl sulfoxide (DMSO) and diluted with DMSO 10% to produce different concentrations (10, 50, 100, 150, 200, 400, 600, and 800 *μ*g/mL). Orlistat was used as a reference for pancreatic lipase inhibition assay and was prepared by the same procedure of plant extract. Pancreatic lipase enzyme stock solution was prepared immediately before use by suspending in DMSO10% at concentration of 1 mg/mL. The stock solution of* p*-nitrophenyl butyrate (PNPB) was prepared according to manufacturer's instructions (20.9 mg of PNPB in 2 mL of acetonitrile). From each working solution of plant extract, 200 *μ*L was mixed with 100 *μ*L of porcine pancreatic lipase (1 mg/mL), and then the volume was brought to 1000 *μ*L with Tris-HCl solution and incubated at 37°C in water bath for 15 min. After the incubation time, 100 *μ*L of PNPB solution was added. The mixture was again incubated in water bath for 30 min at 37°C. A negative control solution was prepared without plant extract, by mixing 100 *μ*L of porcine pancreatic lipase (1 mg/mL) solution with Tris-HCl solution up to 1mL. The same procedure was followed for Orlistat used as positive control. Tris-HCl buffer was used to zero UV-Vis spectrophotometer at 405 nm. Pancreatic lipase activity was determined by measuring the hydrolysis of* p*-nitrophenolate to* p*-nitrophenol at 405 nm using UV-Vis spectrophotometer. The lipase inhibition activity of* M fruticosa serpyllifolia* VOs or Orlistat as a reference was identified by measuring the effect on the enzyme reaction rate after adding extracts, compared with the control. I% was calculated by the using the following equation [37].

(1) $$\begin{array}{c} I\%=\frac{\left[{Absorbance}_{Control}-{Absorbance}_{Test}\right]}{{Absorbance}_{Control}}*100\% \end{array}$$

where I% is the percentage inhibition of pancreatic lipase.

### 2.8. *α*-Amylase Inhibition

The *α*-amylase inhibition assay was done according to procedure conducted by N. Nirmali* et al*. [11] with some modifications. The assay was performed using the 3,5-dinitrosalicylic acid (DNSA) method.* M fruticosa serpyllifolia* VOs stock solution (S.S) of 1 mg/mL was prepared in a minimum amount of DMSO 10% and was further dissolved in buffer (Na_2_HPO_4_/NaH_2_PO_4_ (0.02 M), NaCl (0.006 M) at pH 6.9). Working solution of concentrations 10, 50, 100, 500, and 1000 *μ*g/mL was prepared using buffer (Na_2_HPO_4_/NaH_2_PO_4_ (0.02 M)), NaCl (0.006 M) at pH 6.9). Acarbose was used as a reference. The stock and working solutions of Acarbose were prepared using the same procedure of* M fruticosa serpyllifolia* VOs. *α*-Amylase solution (2 unit/mL) was prepared by dissolving 12.5 mg of amylase enzyme in buffer [(Na_2_HPO_4_/NaH_2_PO_4_ (0.02 M)), NaCl (0.006 M) at pH 6.9]. A volume of 200 *μ*L of *α*-amylase solution (2 unit/mL) was mixed with 200 *μ*L of each VOs working solution and incubated for 10 min at 37°C. Then 200 *μ*L of the starch solution was added and incubated for 3 min. The reaction was stopped by the addition of 200 *μ*L DNSA reagent and boiled for 10 min in a water bath at 85–90°C. The mixture was cooled to ambient temperature and diluted with 5 mL of distilled water, and the absorbance was measured at 540 nm using a UV-Vis. spectrophotometer. The blank with 100% enzyme activity was prepared by replacing the plant extract with 200 *μ*L of buffer. Acarbose was used as a positive control sample. The *α*-amylase inhibitory activity was expressed as percent inhibition and was calculated using (2). The %  *α*-amylase inhibition was plotted against the extract concentration and the IC_50_ values were obtained from the graph [11].

(2) %  α−amylase  inhibition=AbsControl−AbsTestAbsControl∗100%

where %  *α*-amylase inhibition is the percentage inhibition of amylase.

### 2.9. Statistical Analysis

Statistical analysis was conducted using one-way ANOVA with post hoc Tukey-Kramer HSD multiple comparison calculation; *p* values less than 0.05 or 0.01 were considered statistically significant [38].

## 3. Results

### 3.1. Chemical Composition

Volatile oils of the three samples of* M fruticosa serpyllifolia* were extracted using Microwave-Ultrasonic Apparatus, and the produced oils were viscous, colorless, and with a nice peppermint smell. The average percentage of VOs yield (w/w%) of the three samples was Nablus (0.67% ± 0.29), Ramallah (0.99% ± 0.55), and Hebron (0.70% ± 0.17) (Table 1). The data were expressed as mean ± STDV (n=3).

The chemical analysis conducted using GC-MS characterized the VOs with seven components classified into oxygenated ingredients mainly ketones and nonoxygenated ingredients mainly hydrocarbons in all three samples with different proportions (Table 1). The most abundant components in all three samples were pulegone and isomenthone. The total identified components in the three samples were almost consistent in which 90.48, 94.44, and 93.55% of the constituents were identified in Nablus, Ramallah, and Hebron districts, respectively. Detailed results are represented in Table 1. Other five common compounds identified in all three samples with total percentage less than 2% were D-Limonene, *β*-Pinene, Isocaryophyllene, *α*-Pinene, and *β*-Myrcene (Table 1).

### 3.2. Lipase Inhibition Activity

**Figure 1:**
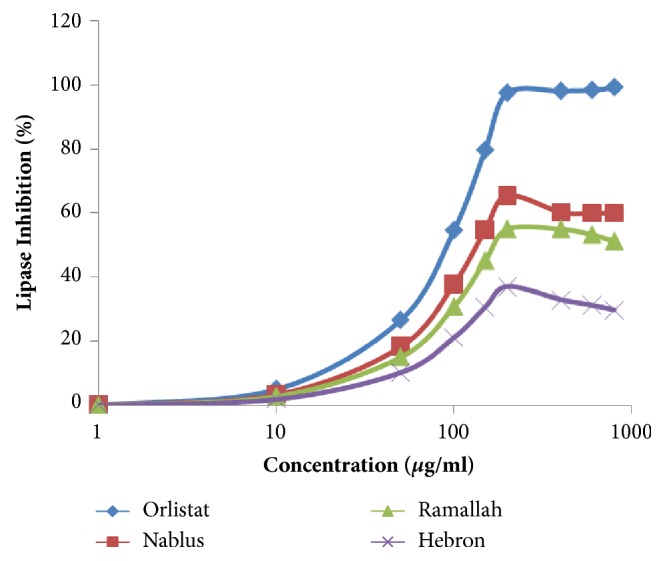
Lipase inhibition assay of the three samples of* M fruticosa serpyllifolia* VOs and Orlistat.

The hydrolysis of* p*-nitrophenyl butyrate to* p*-nitrophenol was used to measure the influence of* M fruticosa serpyllifolia* VOs of three samples on pancreatic lipase enzyme. The assay was detected by comparing to Orlistat lipase inhibitory agent; the three VOs samples showed varied antilipase activity while Nablus VO sample showed the highest potency with IC_50_ value of 39.81 *μ*g/mL but with a maximum inhibition % of 65.40%. However, Orlistat owned potency at IC_50_ value of 43.64 *μ*g/mL with antilipase inhibition of 99.13%. The results of IC_50_ values and the antilipase activity of the three samples and Orlistat are shown in Table 2 and Figure 1. Comparative statistical analysis of the findings of the three samples of VOs showed that there were significant differences in antilipase potency and efficacy of VOs compared to Orlistat (*p* < 0.01). In addition, there were significant differences in antilipase potency and efficacy of VOs compared to each other (*p* < 0.01).

### 3.3. *α*-Amylase Inhibition Assay

**Figure 2:**
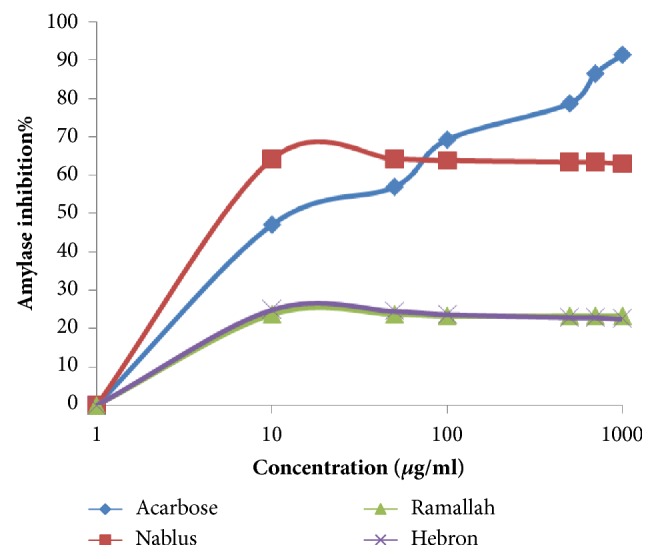
*α*-Amylase inhibition assay of* M fruticosa serpyllifolia* VOs from different regions of Palestine.

*In vitro* assay of alpha amylase inhibitory activities by using starch as a substrate and Acarbose as a positive control was conducted on* M fruticosa serpyllifolia* VOs of the three samples. Our findings revealed that the three samples of VOs showed different degree of inhibition. The highest potency and efficacy of the antiamylase activity of the VOs were reported in the sample collected from Nablus (north), where an IC_50_ value of 3.31 *μ*g/mL was estimated with antiamylase inhibition of 64.34%. The VOs samples from Ramallah and Hebron showed little amylase inhibition activity, while Acarbose showed potency at IC_50_ value of 21.38 *μ*g/mL and I% effect of 91.39%. The results of IC_50_ values and the antiamylase activity of the three samples and Acarbose are illustrated in Table 3 and Figure 2. The three VOs samples showed higher potency in *α*-amylase inhibition compared to Acarbose. There were significant differences in antiamylase potency and efficacy of VOs compared to Acarbose and compared to each other (*p *< 0.01).

## 4. Discussion

The qualitative and quantitative differences in the chemical composition of VOs might be attributed to several factors such as geographical factors (location), climatic effects of the plants, harvest season, nature of the soil, age of the plant parts (young or adult), and time of collection. In addition, the effect of the environment on the secondary metabolic profile of* Micromeria fruticosa* is a model for environmental metabolomics of plants. The north region represented by Nablus city showed the highest percent in *α*-Pinene, *β*-Pinene, and D-Limonene. The middle region represented by Ramallah city owned the highest percent in Pulegone, and the south region represented by Hebron city showed the highest percent in *β*-Myrcene, Isocaryophyllene, and Isomenthone and in total nonoxygenated constituents. These cities are calcified into different biogeographical zones in West Bank in Palestine, such as the central highlands, and the eastern slope which has influence on climate, elevation, the average rainfall, and temperature that affect the rate of biochemical reaction and the weathering process of soil.

The yields of* M fruticosa serpyllifolia* VOs in the current study were lower than that the findings of a study conducted in Palestine studied by Shehab* et al*., [24] which reported a yield of VOs of 2.2%. Also our data were lower in yield than that of* M fruticosa serpyllifolia* growing in Turkey examined by Gulluce* et al*., [28] who reported a yield of 1.85% of VOs of the plant collected in the flowering period.

The GC-MS analysis identified seven compounds listed in Table 1. Studies were conducted previously on* M fruticosa serpyllifolia *VOs.  Table 4 summarizes different relevant literature with the most important finding. In Palestine Shehab* et al*. (2012) reported that pulegone, neo-Menthol, and Isomenthone were the dominant compounds [24], while in Lebanon Pulegone and D-limonene were the prevalent components (Al-Hamawi* et al*., 2011) [13] and for that growing in Turkey piperitenone, pulegone, and Isomenthone were the most abundant components (Gulluce* et al*., 2004) [28]. Isa Telci1 and Mustafa Ceylan (2007) [26] reported that the VOs of subspecies of* Micromeria fruticosa *belong to different chemotypes, (a) pulegone, linalool, and* p*-menthone and (b) piperitenone and linalool type, and revealed that pulegone was the most prominent compound in* Micromeria *species mainly in* M fruticosa*. In the current study Isocaryophyllene was lower than that identified in VO sample of Palestine (Table 4) [24]. The rest of the components in recent study such as D-limonene, *β*-pinene, and *α*-pinene were presented in higher levels than that of the samples of the Palestinian sample and of the Turkey sample. *β*-Myrcene was not identified in the Turkey sample [24, 28]. Pulegone, limonene, *α*-pinene, *β*-pinene, and *β*-myrcene were also being identified in* M*.* barbata* growing in Lebanon but in different proportions [39, 40]. The differences in the total percentages of yields, identified components, and chemical compounds may be explained by the variations in environmental conditions including location, climate, and seasonal and geographical factors [24]: the part of the plant studied and the growing period of leaves; the younger leaves were investigated to be rich mainly in pulegone accounting for 70% of the VOs [19]; it accounted for 29.19% of VOs in the flowering stage [28] and 58.5% before flowering period.

The* p*-nitrophenyl butyrate (PNPB) assay was used as in vitro approach to investigate the inhibition of pancreatic lipase (PL) and to screen the antilipase properties of* M fruticosa serpyllifolia* VOs samples from different regions of Palestine. To the best of our knowledge, there were no previous studies conducted to explore the activity of* M fruticosa serpyllifolia* VOs against PL enzyme. The results of the current study showed that VO sample from Nablus owned the highest potency with IC_50_ value of 39.81 *μ*g/mL (Table 2, Figure 2). However, through an* in vitro* screening of the phytochemical properties of 30 plants growing in Mexico, Villa-Ruano* et al*. (2013) [41] concluded that plants rich with terpenes and other phytochemicals (steroids, tannins, and glycoside) showed very strong antilipase activity. Other studies reported that plant extracts rich in terpenes showed antilipase activity [42, 43]. Investigating* Pinus massoniana* L. volatile oil growing in China by Wang M* et al*. (2017) [44] indicated that the dominant components were related to monoterpene and sesquiterpene (*α*-pinene, *β*-pinene, D-limonene, and caryophyllene) and were responsible for antilipase activity of the oil at IC_50_ 25.10 ±0.49 *μ*M.  The phytochemical screening of the three samples of* M fruticosa serpyllifolia* VOs supports the existence of monoterpenes and sesquiterpenes in all of three samples of VOs. Samples collected from Nablus owned the highest percentages of (*α*-pinene, *β*-pinene, and D-limonene) which support the highest antilipase potency and efficacy of Nablus samples. The antidiabetic properties of* M fruticosa serpyllifolia* VOs samples from different regions in Palestine were investigated by the inhibition of *α*-amylase activity. According to our knowledge, there were no previous studies conducted for the purpose of assessing the activity of* M fruticosa serpyllifolia* VOs against *α*-amylase enzyme. The inhibition of *α*-amylase activity of* Sideritis galactica *Bornm VOs sample growing in Turkey studied by Zengin* et al*. (2016) [45] was related to abundance of monoterpene hydrocarbons ingredients mainly *α*-pinene and *β*-pinene. In screening the *α*-amylase inhibitory activity of* J. phoenicea *volatile oil growing in Tunisia, the results showed powerful *α*-amylase inhibition properties due to presence of terpenes like *α*-pinene [37]. The* M fruticosa serpyllifolia* VOs sample from Nablus owned the highest amount of *α*-pinene and *β*-pinene components (0.91 and 1.48%, respectively) compared with samples of Ramallah and Hebron which may explain its highest potency against *α*-amylase enzyme.

Since the volatile oils of* Micromeria fruticosa serpyllifolia* have a potential inhibitory activity against pancreatic lipase and *α*-amylase enzymes, it is of high importance to take into consideration pulegone toxicity. The European Medicines Agency (EMA), committee on herbal medicinal products (HMPC), concluded that pulegone is considered a hepatotoxin; depending on that, the recommended daily dose of pulegone for 60 kg person by EMA would correspond to 2.3 mg/kg body weight (bw) [46] taking into account the density of pulegone (0.9346 g/mL) [47], and the daily recommended dose as mentioned above could be recommended by specialists as the safe volume of volatile oil ingestion [48].

## 5. Conclusions

*M fruticosa serpyllifolia *VOs from different regions in Palestine represented by three cities showed variable antilipase and antiamylase activities depending on the phytochemical constituents of the volatile oils.* M fruticosa serpyllifolia *VOs of three regions owned the same chemical components but in difference proportions. The sample from north Palestine (Nablus) exhibited highest antilipase and antiamylase activity due to higher amount of *α*-pinene and *β*-pinene. Further* in vivo* studies are needed to evaluate the potential pharmacological activities and to assess the safety and toxicity of plant extract. Also further studies are required to isolate the basic components responsible for potential pharmacological activities.

## Figures and Tables

**Table 1:** The total % of yields, chemical compounds, total identified compounds, and chemical groups of the three samples of *M fruticosa serpyllifolia *VOs.

	%** total VO **	%** total VO **	%** total VO **
	**Nablus**	**Ramallah**	**Hebron**
**(w/w) **%** yield**	0.67% ± 0.29	0.99% ± 0.55	0.70% ± 0.17

**α** **-Pinene**	0.91	0.71	0.83

**β** **-Pinene**	1.48	0.94	1.08

**β** **-Myrcene**	< 0.04	0.26	0.35

**D- Limonene**	1.73	1.65	1.26

**Isocaryophyllene**	0.26	1	1.19

**Isomenthone**	3.16	3.84	14.41

**Pulegone**	82.94	86.04	74.43

**Total non-oxygenated constituents**	4.38	4.56	4.71

**Total oxygenated constituents**	86.1	89.88	88.84

**Total identified components **%	90.48	94.44	93.55

**Table 2:** Lipase inhibition assay of the three samples of *M fruticosa serpyllifolia* VOs and Orlistat.

	**Orlistat**	**Nablus**	**Ramallah**	**Hebron**
**IC**_**50**_ ***μ*g/mL**	43.64	39.81^a^	43.73^ab^	51.21^abc^

**Antilipase activity**	99.13%	65.41%^a^	54.94%^ab^	36.92%^abc^

^a^ *p* < 0.01 compared to Orlistat, ^b^*p*< 0.01 compared to Nablus sample, and ^c^*p*< 0.01 compared to Ramallah.

**Table 3:** *α*-Amylase inhibition assay of the three samples of *M fruticosa serpyllifolia* VOs and Acarbose.

	**Acarbose**	**Nablus**	**Ramallah**	**Hebron**
**IC**_**50**_ ***μ*g/mL**	21.38	3.31^a^	3.40^ab^	3.35^abc^

**antiamylase activity**	91.39%	64.34%^a^	23.77%^ab^	25.00%^abc^

^a^ *p* < 0.01 compared to Acarbose, ^b^*p* < 0.01 compared to Nablus, and ^c^*p* < 0.01 compared to Ramallah.

**Table 4:** Main components and their structures of *M fruticosa serpyllifolia* VOs from different origin.

**Origin **	**Sample period**	**Extraction method**	**Compound and concentration**	**Reference **
Palestine	before flowering stage (March)	hydrodistillation	pulegone 58.5%	Shehab *et al*., (2012) [24]
			neoiso-Menthol 8.7%	
			Isomenthone 3.9%	
			Isocaryophyllene 3.9%	

Turkey	flowering stage	hydrodistillation	piperitenone 50.61%	Gulluce *et al*., (2004) [28]
			pulegone 29.19%	
			Isomenthone 3.92%	

Lebanon	full flowering stage in July	hydrodistillation	Pulegone 30.41%	Al-Hamawi *et al*.,(2011) [13]
			D-limonene 15.64%	
			Menthalactone10.28%	

## Data Availability

The data used to support the findings of this study are available from the corresponding author upon request.

## Supplementary Materials

Supplementary Materials Chemical structure of the main components of* M fruticosa serpyllifolia* VOs. GC-MS chromatograms and analysis.
