# Supplementary material for: Chemical Composition and Enzymatic Screening of Micromeria fruticosa serpyllifolia Volatile Oils Collected from Three Different Regions of West Bank, Palestine
Source: Biomed Res Int. 2018 Oct 16;2018:6536919. doi: 10.1155/2018/6536919 (PMC6206512; doi:10.1155/2018/6536919)
Supplement: Supplementary Materials — Chemical structure of the main components of M fruticosa serpyllifolia VOs. GC-MS chromatograms and analysis. [file 6536919.f1.pdf]

Supplementary data

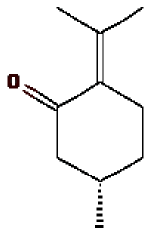

Pulegone [51]

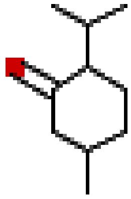

Isomenthone [52]

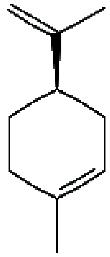

D-Limonene [53]

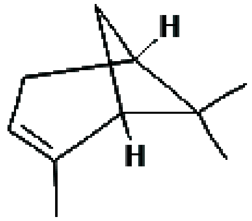

Alpha-Pinene [54]

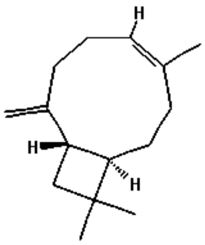

Isocaryophyllene [55]

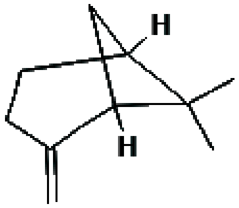

Beta-Pinene [56]

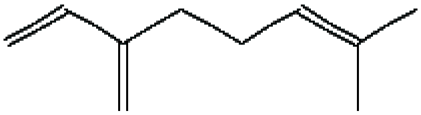

β-Myrcene [57]

Chemical structures of the main components of *M fruticosa serpyllifolia* VOs

Supplementary 2

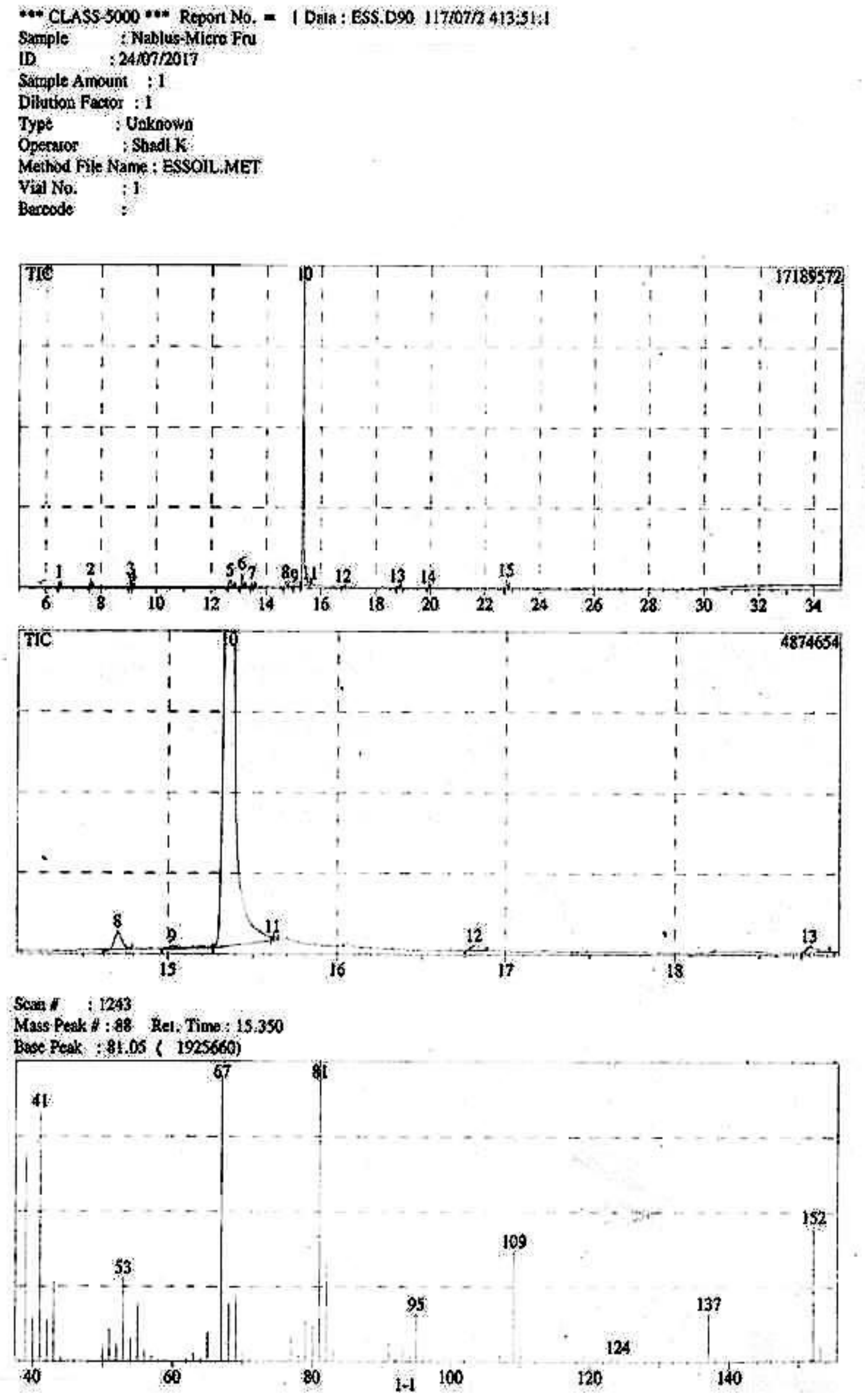

Chromatogram *of M fruticosa serpyllifolia* VO of Nablus

Supplementary 3

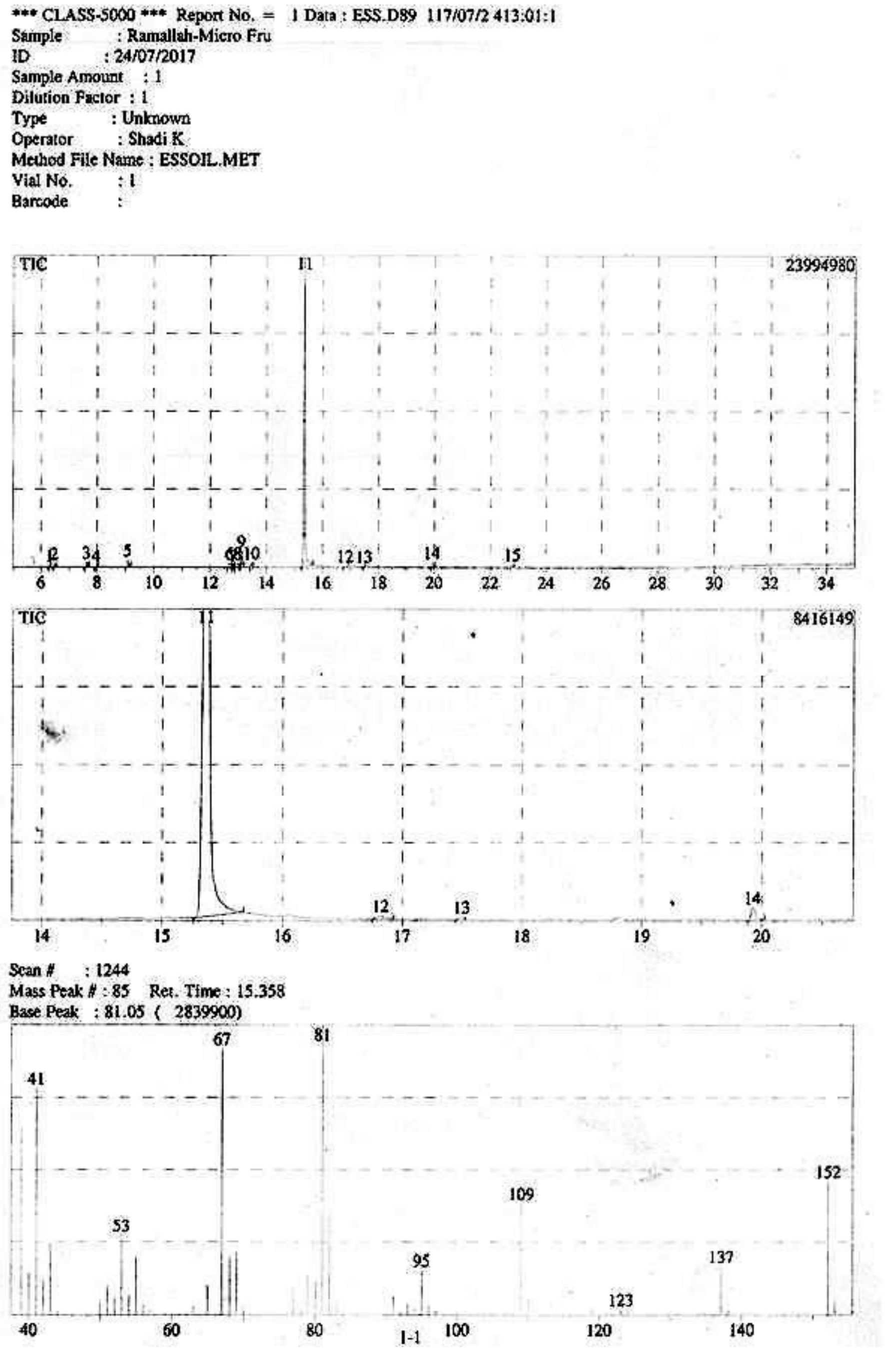

Chromatogram of *M fruticosa serpyllifolia* VO of Ramallah

Supplementary 4

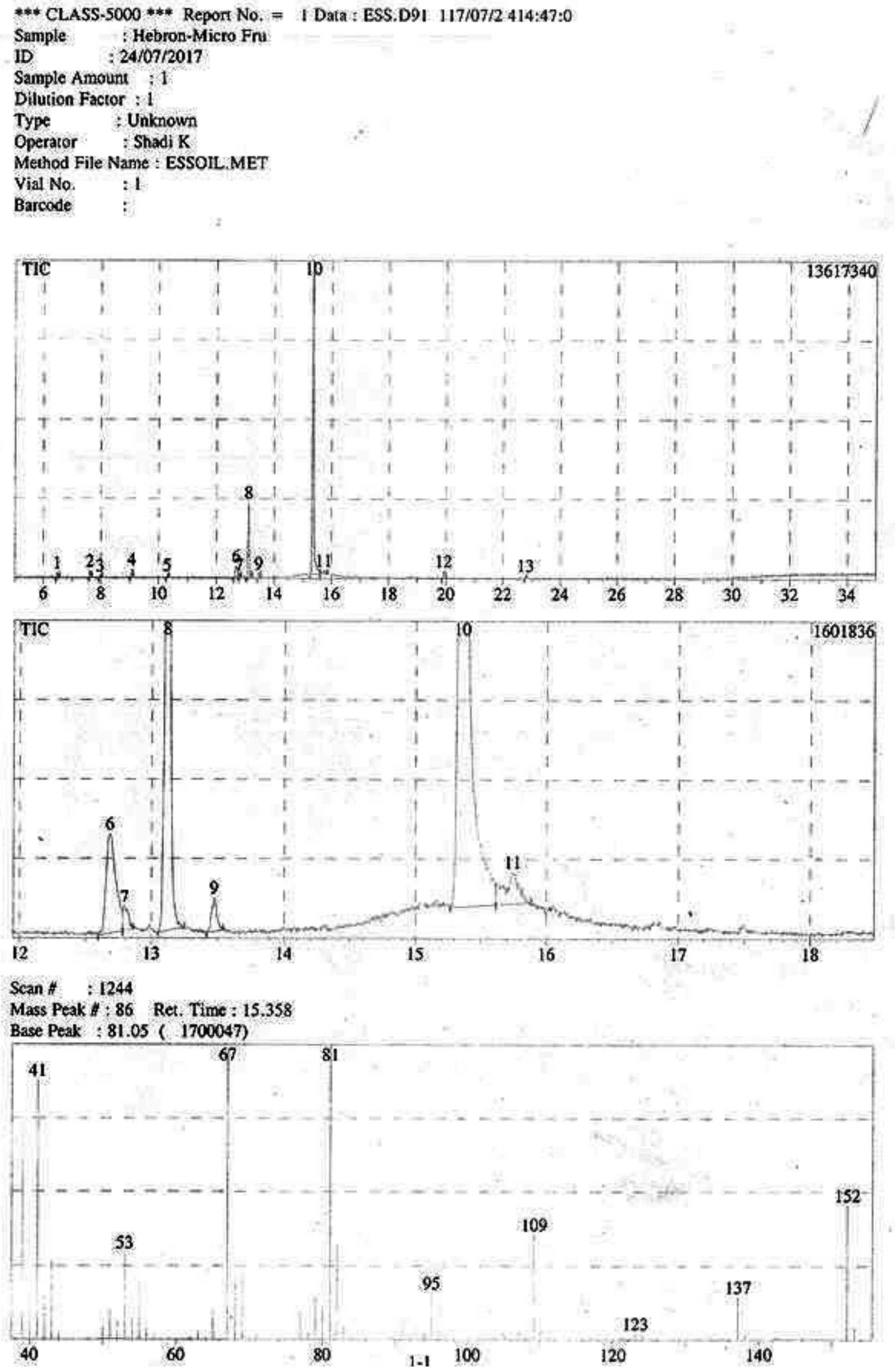

Chromatogram of *M fruticosa serpyllifolia* VO of Hebron
